# How beneficiaries see complex health interventions: a practice review of the Most Significant Change in ten countries

**DOI:** 10.1186/s13690-021-00536-0

**Published:** 2021-02-08

**Authors:** Kendra Tonkin, Hilah Silver, Juan Pimentel, Anne Marie Chomat, Ivan Sarmiento, Loubna Belaid, Anne Cockcroft, Neil Andersson

**Affiliations:** 1 CIET-PRAM, Department of Family Medicine, McGill University, Montreal, Canada; 2 Centro de Investigación de Enfermedades Tropicales (CIET), Universidad Autónoma de Guerrero, Acapulco, Mexico

**Keywords:** Narrative methods, Complex interventions, Participatory research, Intervention evaluation, Most significant change

## Abstract

**Background:**

The Most Significant Change is a story-based evaluation approach used in many international development programs. This practice review summarises practical experience with the approach in complex health interventions in ten countries, with the objective of making it more accessible in evaluation of other complex health interventions.

**Results:**

Participatory research practitioners and trainees discussed five themes following brief presentations by each of the seven attendees who led the exercise: (i) sampling and recruitment; (ii) phrasing the questions to elicit stories; (iii) story collection strategies; (iv) quality assurance; and (v) analysis. Notes taken during the meeting provided the framework for this article.

Recruitment strategies in small studies included universal engagement and, in larger studies, a purposive, systematic or random sampling. Meeting attendees recommended careful phrasing and piloting of the question(s) as this affects the quality and focus of the stories generated. They stressed the importance of careful training and monitoring of fieldworkers collecting stories to ensure full stories are elicited and recorded. For recording, in most settings they preferred note taking with back-checking or self-writing of stories by story tellers, rather than audio-recording. Analysis can combine participatory selection of a small number of stories, deductive or inductive thematic analysis and discourse analysis. Meeting attendees noted that involvement in collection of the stories and their analysis and discussion had a positive impact for research team members.

**Conclusions:**

Our review confirms the plasticity, feasibility and acceptability of the Most Significant Change technique across different sociopolitical, cultural and environmental contexts of complex interventions. Although the approach can surface unexpected impacts, it is not a 360-degree evaluation. Its strength lies in characterising the changes, where these happen, in the words of the beneficiaries. We hope this distillation of our practice makes the technique more readily available to health sector researchers.

## Background

Challenges of evaluating complex health interventions are prominent in current public health debates. Evaluations may be hasty, incomplete [1] or biased towards successful interventions [2]. Complex health interventions are all context dependent, yet many evaluations fail to take account of local knowledge and experience [3]. Narratives can capture local experience, providing a “bridge between data-derived facts and local contextual knowledge” [4].

Narrative evaluation is not a new field, and there is eloquent work on ‘active voicing’ [5] and the objectification of personal experience [6, 7]. The approach is a continuing subject of debate and development, especially the question of whose voice, its representation (format as words, poetry, pictures, and so forth) and who ascribes meaning [8, 9]. It remains a truism that “we naturally talk about ourselves and our lives in a storied way, and we can learn about our lives from these stories” [10].

Development research [11, 12], conservation [13, 14] and education [15–17] have integrated local narratives into evaluation for decades. The Most Significant Change (MSC) technique can document how people experience complex interventions [18]. This approach to narrative evaluation arose from frustrations with conventional evaluation which uses prescriptive criteria to verify achievement of objectives. In contrast, MSC collects stories describing the changes that the intervention beneficiaries experience. This can help to understand *how* the intervention works, whatever its complexity, informing the evaluation and making subsequent interventions more relevant to their local contexts [19]. Conventional evaluation seldom looks beyond simple accountability, like budget disbursement and attainment of stated objectives. Narrative evaluation reaches beyond accountability to be concerned with learning and transformation – how the beneficiaries live the intervention [20]. Narrative evaluation and its collective analysis can itself contribute to positive change among the participants involved and the fieldworkers who collect their stories [21, 22].

There are few reports of MSC in evaluation of health interventions and these deal with very specific situations [23, 24]. This practice review summarizes health sector experience with MSC in participatory initiatives in ten different settings with the objective of making this flexible and inexpensive technique more accessible for evaluation of complex health interventions.

## Methods

In January 2019, the Centro de Investigación de Enfermedades Tropicales and Participatory Research at McGill convened a meeting in Montreal, Canada. The meeting shared experience of the Most Significant Change technique and discussed recommendations for a generalizable method. Attendees included seasoned researchers with decades of MSC experience in Africa and the Americas, including Canada. It also included graduate students working on their first research projects.

Seven participants, all included as co-authors, presented their experience in ten countries to explore MSC in complex health interventions. Meeting attendees considered five methodological issues including recruitment, the questions to elicit stories, story collection, quality control and analysis. All decisions about recommendations were taken by consensus. Two meeting attendees (KT and HS) transcribed notes they took during the meeting. They supplemented these by contacting participants after the meeting. With a third attendee (JP), they conducted a hybrid thematic analysis to the meeting report [25]. The analysis used the initial themes with inductive subthemes emerging during the analysis.

## Results

Meeting attendees included physicians, epidemiologists, public health specialists and graduate students in family medicine and primary care. Their experience of the MSC technique covered 805 stories (Table 1).

**Table 1:** Most significant change evaluations of public health research interventions

Name of project	Country	Intervention	Study design	Intervention participants	Number of stories	Sample	Recording method	Analysis
1. AIDS prevention for the choice disabled (2008–2012)	Botswana Namibia and Eswatini	Structural intervention for primary HIV prevention among vulnerable young women	Cluster randomized controlled trial	75 communities (25 in each country) randomized to one or more of three interventions or control	89 BVV 76 FW	Purposive by country	Notes by story collectors	Hierarchical Deductive thematic
2. Proyecto *Buena Semilla* (2013–2019)	Guatemala	Women’s Circles to empower women as agents of change in their lives, families and communities	Parallel group non-randomized pilot study	> 230 in deliberative dialogue > 550 in Women’s Circles > 60 community health workers and traditional midwives	207	Purposive	Audio-recording by trained interviewer	Deductive and inductive thematic
3. Video edutainment & maternal outcomes in Toro, Nigeria (2015–2020)	Nigeria	Universal home visits to pregnant women and their spouses	Cluster randomized controlled trial in stepped wedge design	Six wards in one local government authority. 33,000 households with population of 260,000.	23 women 21 men 14 visitors 6 govt officers 64 total	Purposive	Notes by story collectors	Hierarchical Deductive and inductive thematic
4. Inter-ministerial National Structural Intervention Trial 2014–2018	Botswana	Structural intervention to reduce HIV infections, particularly among marginalized young women	Cluster randomized controlled trial	Five intervention districts, total population of about 275,000	117 young women	Purposive	Notes by story collectors	Inductive thematic
5. Camino Verde dengue trial (2008–2015)	Mexico & Nicaragua	Community mobilization for dengue prevention	Cluster randomized controlled trial	18,838 households in Nicaragua and Mexico with a total population of 85,182 residents	16 facilitators	Purposive	Self-recorded	Grounded theory; discourse analysis
6. Safe birth in cultural safety (2015–2017)	Mexico	Support for traditional midwives including stipend, apprentices, cultural brokers	Cluster randomized controlled trial	8000 households > 28 traditional midwives > 29 midwife apprentices > 17 intercultural brokers	84	Purposive	Notes by story collectors	Reflexive thematic
7. Pilot cultural safety training (2016)	Colombia	Participation in community-based cultural safety training program	Qualitative descriptive study	13 final-year medical students	13	Universal	Self-recorded	Inductive thematic
8. Training in evidence-based planning (2015–2016)	Botswana: participants from 14 SADC countries	Participation in course on evidence-based planning, including session on MSC technique	Course practical	64 Health planners and researchers (4 classes)	64	Universal	Notes by fellow students in pairs	Inductive thematic
9. Safe birth in post-conflict setting (2019–2020)	Uganda	Co-design of safe-birth intervention	Cross sectional study	3 service providers, 3 men, 3 women, 2 female youth, 3 male youth, 3 CHWs, 3 traditional midwives	20	Purposive	Notes by male and females story collectors	Inductive thematic
10. McGill Participatory Research course (2016–2018)	Canada	Course on Participatory Research, including session on MSC	Course practical	75 graduate students (3 classes)	75	Universal	Self-recorded	Inductive and deductive thematic

*SADC* Southern Africa Development Community. Countries represented on the courses: Botswana, Lesotho, Malawi, Mauritius, Namibia, Seychelles, Swaziland, Tanzania, Zambia and Zimbabwe (Anglophone); Democratic Republic of Congo and Madagascar (Francophone); Angola, and Mozambique (Lusophone)

### Sampling & recruitment

MSC in complex health interventions can differ from conventional qualitative methods on matters of sampling. The technique focuses on the experience of change where this happens, so recruitment to meet this objective involves important biases. MSC is rarely a comprehensive evaluation of the intervention. Sampling focuses on those who *have* experienced the intervention, and who can best describe what worked. In their use of MSC, the projects represented at this meeting did not aim to describe the average or usual effect of an intervention. They gathered accounts of positive or negative change (or no change) to describe what the intervention can do or had done for intended beneficiaries.

The starting point for any evaluation is who contributes evidence or, in this case, who tells the stories. For smaller interventions, it may be possible to collect narratives from all participants. In the pilot project on culturally safe medical education in Colombia [26], for example, all 13 medical students participating in the intervention contributed stories of change (Table 1, project 7).

Large-scale or multi-site interventions present representation challenges and selection sets the limits of interpretation. Most projects discussed during the meeting used purposive sampling to select story tellers. In most cases, fieldworkers purposively selected story tellers they thought might have benefited from the intervention. In the Botswana HIV prevention project, fieldworkers who facilitated workshops selected young women to tell stories (Table 1, project 4). In Nigeria (Table 1, project 3), fieldworkers who did home visits visited women and men who could provide narratives of change. This approach tries to highlight the experience of change. It says little about the proportion who experience the change or about those who do not experience it. In Uganda, the coordinator and community midwife selected MSC contributors from participants in project activities (fuzzy cognitive mapping, focus group discussions, and deliberative dialogue), with stories elicited from each of several defined stakeholder groups (women, men, youth, traditional midwives, community health workers, and service providers) (Table 1, project 9).

Sampling can be stratified to ensure representation of specific intended beneficiaries. This strategy provides information on similarities and variability between stakeholders. Researchers in the trial on safe birth in Mexico (Table 1, project 5) collected stories from traditional midwives, their apprentices, and intercultural brokers. The sampling within each stratum can be purposive, systematic or random.

### Phrasing the question(s)

Attendees noted the perennial challenge to ensure narrators recount the full stories. Careful supervision can avoid getting just a few words or sentences from an intervention participant describing what they consider an important change. Storytelling is a ubiquitous and transcultural practice. Yet to bring this into play, gathering narratives from intervention participants requires careful phrasing of the question(s). The wording of the question or questions matters.

Attendees cautioned against literal translation of MSC questions into the local language. This can cause confusion. Attendees advocated for cultural adaptation of questions in consultation with local stakeholders. The safe birth study in Mexico (Table 1 project 6), for example, had to address the absence of the word ‘significant’ in the local Indigenous languages. The standard MSC question would have been ineffective for stories about the impact of the intervention on their lives. On local advice, they narrowed the scope of the enquiry. They adapted the question to elicit positive change, to ask “*Can you share a story with me that makes your heart feel happy?*” Different wording might have limited the bias toward positive stories implicit in this question.

Most projects used more than one question to prompt the recounting of complete stories. An icebreaker question at the beginning of the story collection can help story tellers to feel more comfortable. In Mexico, the facilitator shared the icebreaker *(“How have you felt during the project?”*) with the whole group so all the participants heard each other’s responses. Then a question to each respondent asked for a real-life story and another question asked why the story meant the most to them. The initial question focused attention on the story. The second allowed story tellers to share their feeling about the change in a more abstract way. In the Guatemala project, the questions tried to collect stories of both individual and collective change. In the Ugandan project, the first question explored with story tellers how participation in the project activities affected their lives. The second question invited them to illustrate this through a personal story. Some projects used a third question about what respondents would like to change in a future scenario. This counterbalanced the emphasis on positive experiences that characterizes MSC.

Some projects, for example in Nigeria, included extra instructions to emphasise the goal of capturing real-life stories. Others requested narrative details about places, dates or characters to assure verisimilitude. In all cases, researchers informed narrators the story would have no impact on their participation in the project or its benefits.

An extra question to story tellers could provide the basis for economic analysis. “*How much was that change worth to you?*”. A cash amount or some interpretable equivalent, like work time, puts a value on the change. This can contribute to a stakeholder-informed social return on investment analysis. It can also be useful in later knowledge translation strategies [27, 28]. Without replacing a cost-benefit analysis, this offers a discussion point on sustainability and scale implementation.

### Story collection and recording

#### Training and monitoring field workers

Meeting attendees emphasized the importance of story collector training to avoid common errors, like recording respondent accounts in a few phrases rather than a full narrative of change. They described training fieldworkers to use probes such as: “*Can you tell me about an event that shows this change?”, “Can you give me some more details?*”. These probes helped to ensure fuller accounts of change. As in all interview research, interviewers should use probes in a mindful way to elicit insights without side-tracking the process.

Attendees gave examples of what they found helped to prepare fieldworkers to collect stories. This included training about common errors, using practical role-play. One participant explained, “*After classroom training, a supervised pilot exercise allowed us to build confidence in eliciting stories and recording them. It also allowed us to identify fieldworkers not capable of carrying out the task. A supervisor should go with story collectors and make periodic quality checks.*”

#### Fieldworkers and narrator power dynamics

Attendees highlighted power dynamics between story-collectors and story-tellers. They agreed on the importance of compatibility between story-collectors and story-teller gender in terms of age, culture, social status, and language. Story collectors must speak the local languages and have good knowledge of their communities. Story tellers need to be able to trust story collectors. Matching storytellers and story collectors by age helps. Each generation has its own sub-cultural and linguistic norms that are not always intelligible to outsiders. Genuine narratives emerge more easily when story-collectors and story-tellers do not have major power differentials.

The *Camino Verde* project in Nicaragua and Mexico added a new dimension by including the MSC in a mid-term peer evaluation. Community members from 75 intervention sites visited each other to collect the stories of their peers. As they discussed the most meaningful stories (see below, Analysis), they shared experience and evaluated each other’s progress. Their shared interests gave the stories added meaning, reaffirming and motivating those who implemented the complex participatory intervention.

#### Methods for recording stories

The researchers described three strategies they had used for recording stories. 1) Audio recording with verbatim transcription. 2) Note-taking during the story, back-checking with the story tellers before a full write-up. 3) Self-written stories by the story tellers themselves. Each method has its advantages and disadvantages. How best to record stories depends on language, literacy of participants and personal preference. The meeting attendees discussed the consequences of recording or writing the stories. Notwithstanding the advantages of electronic recording, the paper option had notable benefits for those who contribute stories. In many communities, it is uncommon to be recorded while speaking. Audio recording can lead to a stilted product or make participants feel insecure about sharing information. The intercultural brokers in Mexico used paper for notes and, in some cases, drawings of their stories. Indigenous participants with limited writing ability enjoyed illustrating their story, increasing their engagement with the exercise.

A few questions can help prospective MSC users to decide how to record stories: Is the language used by the story tellers oral or written? What is the level of literacy of the story tellers, in any language? How would story tellers prefer to record their stories? How experienced are the story-collectors in note taking?

### Methods for data analysis

The meeting reviewed four methods to analyse MSC stories: hierarchical group analysis, thematic analysis, grounded theory and discourse analysis. Regardless of the analysis strategy, attendees recommended sharing results of the story collection with story tellers and other intervention participants and local stakeholders. This is an opportunity for member checking and to promote discussion and participatory action.

#### Hierarchical group analysis

Elaborated and popularized by Dart and Davis, hierarchical group analysis selects stories and filters them through levels of authority within an organization or program, until a final handful of stories reflect the most significant change [9]. Selecting stories at each level takes time. Meeting attendees who had used this approach took at least one full day for each level of selection. They used a standard format to register reasons for the selection of each story at each level. Sharing the stories with the group in advance helped to speed things up. Decision-making processes to choose the best story varied between projects. Some used consensus, others voted and still others a mix depending on participant preferences.

The mid-term evaluation of the three-country Choice Disability trial in southern Africa involved the country teams and central team reviewing the stories. Before the meeting, all participants read all the stories from all three countries. Together they selected the most significant stories from each of the three countries. Each participant identified their chosen story and gave their reasons for their choice. Team members voted on selection of stories from each country. They decided to select more than one story per country. The discussion about the stories helped the team to think about what the intervention was really aiming to achieve. It allowed open discussion of the actual and intended direction. The team found reviewing the stories encouraging and validating. The stories reflected how their work could produce profound improvements in the lives of vulnerable people.

Hierarchical group analysis can offer insights into expected and unexpected effects. It can thus be useful to inform mid-stream adjustments. It can remind the team of what they had intended the intervention to achieve at the outset. Collaborative sessions help to identify stories that raise concerns of credibility. Although this is not the right setting to judge veracity of each story; a standardised recording format can include a space to note this concern.

A limitation of hierarchical group analysis is that informative stories do get discarded. Managers at different levels may value different outcomes and narratives of change, and those might be different again from what intended beneficiaries appreciate. Dart and Davis note that “*there is no reason to restrict story selection to steering committees and investor groups*” [18]. Meeting attendees also reported using other forms of group analysis, such as participants themselves selecting the final stories. This counterbalanced the vertical hierarchy of story selection and gave weight to the priorities of participants.

#### Thematic analysis

Thematic analysis reviews all stories, at least to the point of saturation in the analysis. This allows evaluators to examine different types of changes, independent of the intervention implementation team. Within the constraints of the questions asked and who they were asked of, this can provide a fuller picture of the impact. Thematic analysis hopes to identify what the changes were and how these changes happened. The reasons for choices expressed during preceding collaborative selection can inform thematic analysis. These reasons might differ from the original goals of the project as they include the interests of complex intervention beneficiaries and the people involved in implementation.

Thematic analysis can be inductive, when researchers identify themes emerging in the transcripts or reports of the stories. It can also be deductive when researchers use a pre-established theory to inform their themes. Sometimes it is a hybrid of the two approaches. Meeting attendees had mostly used reflexive thematic analysis, adapted from Braun and Clarke [25]. In this approach, researchers who are familiar with the project objectives and theory of changes build codes and themes from scratch as they review the stories. They then define their meaning, name, and relationships, referring to existing literature and/or a theory of change. They create a theme map to display relationships between concepts. The Mexican team used this approach in the Safe Birth project. The Colombian work with the medical students and the Uganda co-design with marginalised women both used an inductive thematic analysis to define categories of change.

When there is a robust theory of change, this can inform deductive thematic analysis of the MSC stories. The CASCADA results chain [29], for example, proposes a partial order of intermediate outcomes from Conscious knowledge, through Attitudes, Subjective norm, Change intention, Agency, Discussion or socialization, and then Action or behavior change. This can provide a framework for deductive thematic analysis. In the mid-stream evaluation in the three-country Choice Disability trial, researchers looked for these themes in different stories to illustrate progress along this partial order of intermediate outcomes [30]. The Guatemala analysis used themes in its theory of change (social support, self-esteem and communication) built with fuzzy cognitive mapping. In a separate exercise the same team relied on grounded theory (see below).

Another example of deductive thematic analysis, in the case of educational initiatives, is the application of Blooms taxonomy. This looks for verbs or similar wording in each of Bloom’s six levels of learning: 1. Knowledge of previously learned information; 2. Understand the meaning; 3. Apply knowledge; 4. Analyse problems or concepts; 5. Synthesis of elements to create new concepts; and 6. Define evaluation criteria [31].

#### Grounded theory

Key to grounded theory is *not* engaging with the literature or a theory of change before beginning analysis. This avoids shaping the analysis with preconceptions or lexicon from existing research. It allows themes to arise in a genuine way from the data. The Guatemalan project used grounded theory to analyses MSC stories to build their theory of change. They found the same intermediate outcomes recurred in Women’s Circles and Deliberative Dialogue. The researchers listed the outcomes and descriptions of how one led to another. The stories described an initial cultivation of trust and a sense of belonging within the group. Participants said this increased their awareness of others in their community. It helped them to feel more confident in their ability to express themselves and share their opinions. The participation could lead to improved relationships with others (friends, family, partners, colleagues, etc.). It improved emotional health and wellbeing. The Guatemalan case showed reduced substance use and it generated a newfound desire to help others within their community.

Grounded theory also supported analysis of the MSC stories collected in the Mexican arm of the *Camino Verde* trial. Anthropologist Joan Muela found “*learning by doing*” and “*ownership effect*” (*efecto de lo propio*) were emerging themes. Learning about the mosquito’s reproductive cycle combined with repeated visits by volunteers [32].

In its conventional form, grounded theory relies on experienced trained social scientists who can genuinely ignore what they already know or believe, to allow the theory to emerge from the data. An alternative grounded theory could emerge from socialized or participatory analysis, without participants pretending to be ignorant of what they know. Without explicit reference to a theory of change or literature, stakeholders analyze story content to generate a theory based on the data. In this case, the emic perspective provides the theory, even if not enunciated as such.

#### Discourse analysis

Discourse analysis includes different approaches to language or systems of meaning [33]. It treats narrative content almost as one might a new language, looking for the choice of words, their frequency and partial order. The stories from traditional midwives in Mexico, for example, showed a transition over the duration of their involvement in the project. Early stories reflected colonialism and acculturation when they saw their knowledge neglected. Later stories reflected emancipation and cultural pride associated with increased cultural safety.

In participatory complex interventions, we expect personal and social transformations. We treat this transformation as a broadly desirable outcome without expecting to control or even to understand the nature of the transformation. Since it often provokes innovation, participatory research can change lexicon and shift meaning. It can be helpful to understand this underpinning of outcomes. With this, we can fine tune the intervention around it. Discourse analysis helps to understand elements of the architecture of transformation. In Mexico, discourse analysis of MSC stories began with open coding to identify words and sequences that reflect transformation. Theoretical sensitivity around words like “emancipatory” or “empowering” allowed us to see the patterned meaning of these in the data. Since discourse analysis depends on the exact words and partial order of words, it is best to work with written or, when literacy or language are issues, recorded stories. Back translation should be part of any translation strategy, although discourse analysis is likely to be more reliable in the language of the stories.

## Discussion

This practice review summarizes practical experience of five aspects of using MSC in participatory health initiatives in different settings, including recruitment (who contributes stories), the questions asked, mechanics of story collection, quality control and analysis. The Montreal meeting echoed some issues already raised in the MSC literature outside of the health sector [13, 17, 18]. There is already ample recognition of the value of careful training of fieldworkers and the value of regular quality checks during MSC stories data collection [34]. The meeting also revealed field-based insights from using MSC in complex health interventions, particularly on the dynamics of community engagement and participation.

Storytelling is an established tradition in many cultures, which should be a major plus in adopting and adapting narrative evaluation. In research fieldwork, however, storytelling often takes place outside local cultural practices, somehow failing to capitalise fully on the narrative traditions. Attendees were emphatic about the need for practice-based training of fieldworkers in story-collection. They recommended linguistic and cultural adaptation of the questions. They favoured use of multiple questions, probes and regular quality assurance checks. Rather than limiting the evaluation to intended beneficiaries, attendees saw merit in different knowledge users providing and analyzing MSC stories, to collate a full range of accounts of the intervention impacts. Hearing the actual words stakeholders use to describe changes in their lives is a valuable additional product of MSC. One researcher explained the transformative capacity of MSC as “*the process of gathering and analyzing the stories creates change in itself*”.

Attendees also acknowledged the role of MSC in external communications of how the intervention worked. They reported using stakeholder wording in reports, publications and visual media. When MSC is part of a mixed method evaluation of a complex health intervention, the actual words of participants can usefully complement statistics from a quantitative component. Formal thematic analysis of MSC stories can provide depth and dimension to a simple account of impact.

### Limitations

We are aware this small meeting represents only a fraction of the worldwide experience with using MSC in the health sector. The experience presented and discussed at the meeting arose from an established practice of mixed method participatory research. Some pivotal concerns of the meeting attendees, like the transformative value for intervention beneficiaries, might be less of a concern in conventional researcher-led settings.

A formal step in researcher-led MSC is the clarification or setting of ‘domains of change’. These are broad categories by which researchers collect and classify stories [35]. In contrast, participatory research engages stakeholders throughout the process, including contributing narrative evidence. Participatory research also means less emphasis on what *type of stories* to collect. It focuses on *who* contributes stories and accepts their concerns as the concerns of the evaluation. Participatory research is often more relaxed than conventional investigator-led research about prior theories and literature. It relies on local ownership and local knowledge.

Important limitations of the MSC technique [15, 20] are hard to overcome whatever the context of its application. By its very name and focus on change, MSC is not a comprehensive evaluation approach. In the experience of meeting attendees, its strongest use is in understanding the nature of the change when change does happen. Although results can be surprising, they rarely reveal in why change does *not* happen. As a qualitative approach, MSC cannot address issues like coverage by the intervention or the proportion of participants who benefit. Attendees had used MSC as one of several approaches in multi-method participatory research. MSC stories are not always comprehensive summaries of the underlying mechanisms of a transformation. This can be better explored with less specialized skills using fuzzy cognitive mapping [36].

## Conclusion

The meeting summarized diverse international experience of MSC in complex health interventions. The technique helps to clarify the impact of an intervention and its meaning for individual beneficiaries and their communities. It can explore and characterize unexpected changes. The more we understand about impact on people’s lives, the more likely our next interventions will achieve a meaningful impact. The meeting highlighted flexibility and value of the approach in various sociopolitical, cultural and environmental contexts. The application of MSC in both simple and complex interventions will continue to evolve as researchers explore new ways to tell, to record, to analyze, and to use stories.

## Data Availability

All data generated or analysed during this study are included in this article.

## Abbreviations

HIV: Human Immunodeficiency Virus
MSC: Most Significant Change

## References

[1] More L, Hallingberg B, Wight D, Turley R, Segrott J, Craig P, Robling M, Murphy S, Simpson S, Moore G (2018). Exploratory studies to inform full-scale evaluations of complex public health interventions: the need for guidance. J Epidemiol Community Health.

[2] Bonnel C, Jamal F, Melendez-Torres G, Cummins S (2015). ‘Dark logic’: theorising the harmful consequences of public health interventions. J Epidemiol Community Health.

[3] Minary L, Alla F, Cambon L, Kivits J, Potvin L (2018). Addressing complexity in population health intervention research: the context/intervention interface. J Epidemiol Community Health.

[4] Kothari A, Wathen C (2017). Integrated knowledge translation: digging deeper, moving forward. J Epidemiol Community Health.

[5] Robertson M, Moir J, Skelton J, Dowell J, Cowan S (2011). When the business of sharing treatment decisions is not the same as shared decision making: a discourse analysis of decision sharing in general practice. Health.

[6] Gwyn R (2000). “Really unreal”: narrative evaluation and the objectification of experience. Narrat Inq.

[7] Fredrickson BL, Roberts TA (1997). Objectification theory: toward understanding women's lived experiences and mental health risks. Psychol Women Q.

[8] Byrne G (2017). Narrative inquiry and the problem of representation:‘giving voice’, making meaning. Int J Res Method Education.

[9] Larson CL (1997). Re-presenting the subject: problems in personal narrative inquiry. Int J Qual Stud Educ.

[10] McIssac BE (2008). Narrative inquiry: a spiritual and liberating approach to research. Relig Educ.

[11] Kraft K, Prytherch H (2016). Most significant change in conflict settings: staff development through monitoring and evaluation. Dev Pract.

[12] Shah R (2014). Assessing the ‘true impact’ of development assistance in the Gaza strip and Tokelau: ‘Most significant change’ as an evaluation technique. Asia Pac Viewp.

[13] Wilder L, Walpole M (2008). Measuring social impacts in conservation: experience of using the Most significant change method. Oryx..

[14] Reed SO, Friend R, Jarvie J, Henceroth J, Thinphanga P, Singh D, Tran P, Sutarto R (2015). Resilience projects as experiments: implementing climate change resilience in Asian cities. Clim Dev.

[15] Heck D, Sweeney T (2013). Using Most significant change stories to document the impact of the teaching teachers for the future project: an Australian teacher education story. Aust Educ Comput.

[16] Major L, Swaffield S. Experiences introducing the Most significant change technique to support leadership for learning in Ghana. Commonwealth Centre for Education Report no. 14. University of Cambridge. 2014 https://www.educ.cam.ac.uk/centres/archive/cce/publications/CCE%20Report%2014%20LfL%20Ghana%20MSC.pdf Accessed 28 Feb 2020.

[17] Choy S, Lidstone J (2013). Evaluating leadership development using the Most significant change technique. Stud Educ Eval.

[18] Dart J, Davies R (2003). A dialogical, story-based evaluation tool: the Most significant change technique. Am J Eval.

[19] Ørnemark, C. Learning journeys’ for adaptive management–where does it take us. GPSA note 12, global Partnership for Social Accountability. 2016 http://gpsaknowledge.org/wp-content/uploads/2016/04/NOTE_march.pdf (Accessed 1 July 2019).

[20] Willetts J, Crawford P (2007). The Most significant lessons about the Most significant change technique. Dev Pract.

[21] Bandura A (2004). Health promotion by social cognitive means. Heal Educ Behav.

[22] Polet F, Malaise G, Mahieu A, Utrera E, Montes J, Tablang R, Aytin A, Kambale E, Luzala S, Al-Ghoul D, Darkhawaja RA (2015). Empowerment for the right to health: the use of the" most significant change" methodology in monitoring. Health Human Rights J.

[23] Ho LS, Labrecque G, Batonon I, Salsi V, Ratnayake R (2015). Effects of a community scorecard on improving the local health system in eastern Democratic Republic of Congo: qualitative evidence using the most significant change technique. Confl Heal.

[24] Limato R, Ahmed R, Magdalena A, Nasir S, Kotvojs F (2018). Use of most significant change (MSC) technique to evaluate health promotion training of maternal community health workers in Cianjur district, Indonesia. Evaluation Program Planning.

[25] Braun V, Clarke V (2006). Using thematic analysis in psychology. Qual Res Psychol.

[26] Pimentel J, Kairuz C, Merchán C, et al. The experience of Colombian medical students in a pilot cultural safety training program: a qualitative study using the Most significant change technique. Teach Learn Med. 2020:1–9. 10.1080/10401334.2020.1805323. 10.1080/10401334.2020.1805323 32812831

[27] Nicholls J (2017). Social return on investment—development and convergence. Evaluation Program Planning.

[28] Hall M (2014). Evaluation logics in the third sector. Volunt Int J Volunt Nonprofit Org.

[29] Andersson N, Nava E, Sanja M, Beauchamp M. The women made it work: fuzzy transitive closure of the results chain in a dengue prevention trial in Mexico. BMC Public Health 2017;17 (408): 133–139. https://doi.org/10.1186/s12889-017-4301-0. 10.1186/s12889-017-4301-0 PMC5506572 28699563

[30] Cameron M, Cockcroft A, Waichigo G, Marokoane N, Laetsang D, Andersson N (2014). From knowledge to action: participant stories of a population health intervention to reduce gender violence and HIV in three southern African countries. AIDS Care.

[31] Forehand M (2010). Bloom’s taxonomy. Emerging Perspectives Learning Teaching Technol.

[32] Ledogar R, Hernández C, Morales-Perez A, Andersson N (2017). Mobilizing communities for dengue prevention: socialising evidence for participatory action. BMC Public Health.

[33] Schwandt TA (2007). The SAGE dictionary of qualitative inquiry.

[34] Dart J, Davies R. The 'Most Significant Change' (MSC) Technique: A guide to its use. 2005 10.13140/RG.2.1.4305.3606

[35] Reid E, Reid J Story-based impact assessment: outline of a story-telling methodology; PNG. 2004 https://groups.yahoo.com/neo/groups/MostSignificantChanges/info Accessed 28 February 2020.

[36] Andersson N, Silver H (2019). Fuzzy cognitive mapping: an old tool with new uses in nursing research. J Adv Nurs.

